# Spontaneous changes in mandibular incisor crowding from mixed to permanent dentition: a systematic review

**DOI:** 10.1186/s40510-023-00466-3

**Published:** 2023-05-08

**Authors:** Cibelle Cristina Oliveira dos Santos, Renata Travassos da Rosa Moreira Bastos, Silvio Augusto Bellini-Pereira, Daniela Garib, David Normando

**Affiliations:** 1 Department of Orthodontics, Federal University of Pará, Belém, Brazil; 2 Department of Orthodontics, Bauru Dental School, University of São Paulo, São Paulo, Brazil; 3 Department of Orthodontics, Bauru Dental School, University of São Paulo, São Paulo, Brazil; 4 Department of Orthodontics, Dental School, Federal University of Pará, Tv Almirante Wandenkolk, 1243- sala 1503, Belém, Pará 66055-090 Brazil

**Keywords:** Crowding, Malocclusion, Child, Mixed dentition, Orthodontics

## Abstract

**Introduction:**

Dental crowding is the most prevalent malocclusion in the mixed and permanent detitions and can have a major impact on dentofacial esthetics. However, adjustments to the development and growth of the dentition can potentiate self-correction of dental crowding during childhood.

**Objective:**

To evaluate the physiological behavior of mandibular incisor crowding in the transition from mixed to permanent dentition.

**Methodology:**

Five electronic databases (PubMed, Scopus, Web of Science, LILACS and LIVIVO) and part of the gray literature (Proquest and Google Scholar) were investigated, based on the eligibility criteria associated with the acronym PECO, until June 2022. The risk of bias was assessed using the ROBINS-E tool and the certainty of evidence, the GRADE tool.

**Results:**

Among the 2.663 studies identified, five were selected for qualitative analysis, of which one have a low risk of bias, and four, a moderate risk. A total of 243 patients were evaluated. Evidence with a high level of certainty was generated indicating a tendency for improvement in mandibular incisor crowding from mixed to permanent dentition, with mandibular incisor crowding decreasing from 0.17 to 4.62 mm on average. The mandibular incisor crowding reduction seems to be associated with the amount of initial crowding and spontaneous dental arch dimensional changes that occur in the mixed dentition and culminate in the increase in arch perimeter, leeway space, incisor protrusion and transverse growth of the maxillary and mandibular arch.

**Conclusion:**

Based on moderate scientific evidence, spontaneous longitudinal changes in dental arch in the transition from the mixed to the permanent dentition demonstrate a spontaneous improvement in mandibular incisor crowding by up to 4.62 mm. These evidence provide a scientific basis for planning only longitudinal follow-up in patients with mild to borderline moderate mandibular incisor crowding in the mixed dentition avoiding overtreatment.

**Supplementary information:**

The online version contains supplementary material available at (10.1186/s40510-023-00466-3).

## Introduction

The impact on smile esthetics and quality of life [1], associated with a incisor crowding prevalence of 53.4% ​​in mixed dentition [2], encourage many parents to take their children for a dental evaluation [3]. At this occlusal stage, there is some possibility of a spontaneous physiological correction of dental crowding in the region of permanent mandibular incisors. Among the factors related to this adjustment in occlusion are the increase of approximately 3 mm in the intercanine distance [4], the preservation of Nance´s leeway space providing around 4.3 mm in the intramandibular arch dimension [5], and the greater protrusion of permanent incisors compared to the deciduous incisors [5]. The challenge faced by the clinician is to identify to what extent can expect self-correction, and which children will develop definitive or temporary problems due to space deficiency. Even though it is possible to use mixed dentition analyses to predict future intra-arch dimensional changes [6], the variability in the individuals' craniofacial growth can reduce the clinical reliability of these analyses. [7]

The difficulty in predicting the magnitude of spontaneous mandibular incisor crowding changes in the mixed dentition may lead professionals to carry out arch expansion, deciduous canine stripping or dental extraction protocols [8]. However, the literature reports that some therapeutic approaches, such as deciduous canine stripping, can reduce crowding self-correction potential during childhood. [9]

The decision of the clinician to monitoring occlusion development and the evolution of mandibular incisor crowding or to perform early treatment can be influenced by the small impact of the malocclusion on quality of life in children younger than 11 years old [10, 11]. Additionally, subjecting the child to early treatment for long periods, with the risk of pain or discomfort and relying on patient collaboration, can be factors that have a greater impact on the child well-being rather than small crowding of the mandibular permanent incisors.

In this context, the aim of this systematic review is to investigate the spontaneous changes of mandibular incisor crowding from mixed to permanent dentition and thus producing scientific basis for definition of treatment planning in face of mandibular incisor crowding.

## Material and methods

### Protocol and registration

This study was registered in the PROSPERO database, under the number ID:340493 and followed the guidelines of the Preferred Reporting Items for Systematic Reviews and Meta-Analysis [12] (Additional file 1: Appendix S1).

### Eligibility criteria

The inclusion criteria for the study selection were based on the PECOS acronym as follows:

Population (P): Children in the mixed dentition.

Exposure (E): Mandibular incisor crowding.

Comparator (C): Before and after the patient themselves.

Outcomes (O): Changes in crowding in the transition from mixed to permanent dentition.

Study design (S): Observational and clinical studies.

The exclusion criteria consisted of patients with early loss of primary teeth, interproximal caries with loss of intra-arch space, dental anomalies of number or shape and impacted canines. Moreover, conference abstracts, case reports, opinion articles and book chapters were also not considered as eligible criteria.

### Information sources

Five electronic databases were accessed to perform the search strategy: PubMed, Scopus, Web of Science, LILACS and LIVIVO. Gray literature search included Proquest and Google Scholar. Also, hand search was conducted in the reference lists of the included articles in case of missing any relevant study to the research topic. No language restriction was applied, and coverage dates were not limited. The search was conducted until June 6, 2022, and the alerts have been verified until January 26, 2023.

### Search strategy and study selection

The search strategy was elaborated with the combination of MeSH, entry terms and keywords related to the PECO acronym, associated with the use of Boolean operators “OR” and “AND.” The search strategy for each database is presented in Additional file 2: Appendix S2. All the relevant citations were exported to a bibliography reference manager software (EndNote, × 9 version, Clarivate Analytics, Philadelphia, PA, USA) where the duplicate references were excluded. Two independent reviewers (C.S. and R.B.) selected the included articles in two phases. In phase one, the titles and abstracts were evaluated considering the eligibility criteria. In phase two, the full texts of the potentially relevant studies were assessed and selected following the same criteria as in phase one. Then, all the information found was crosschecked. In case of any disagreements, the third and fourth authors (S.B.P. and D.N) were consulted before a final decision was made in both phases. If important data for the review were missing or unclear, the corresponding author of the included study was contacted to resolve or clarify the concern.

### Data extraction

The same authors from the search phase independently collected the data from the selected articles. All the data were extracted manually using an excel spreadsheet. Once selected, the retrieved information was crosschecked with the third reviewer (S.B.P), when necessary. The extracted information was related to authorship (author, year of publication and study design), characteristics of patients (sample size, sex, follow-up period, and mean age), clinical characteristics (diagnostic method of crowding, secondary evaluations and statistical analysis), main results (mean value of crowding in mixed and permanent dentitions, change in crowding in the transition of dentition) and conclusion. The extracted data are identified in Table 1.

**Table 1:** Summary of the data from included studies

Author, year. Study design Country	*n* (M/F) Mean age (SD) follow-up	Mandibular incisor crowding diagnosis Secondary evaluations Statistical analysis	Results						Conclusions
			Mandibular incisor crowding in the mixed dentition (M/SD)		Mandibular incisor crowding in the permanent dentition (M/SD)		Mandibular incisor crowding change in the transition of dentition (M/SD)		
Sinclair and Little [19] Retrospective cohort study USA	65 (33/32) 9.06 years 4 years	Little index Intra- and inter-arch measurements Pearson's correlation and *t*-test	2.22 (1.23) mm		2.00 (1.17) mm		-0.17 (1.75) mm		Although the average amount of mandibular incisor crowding reduction is only 0.17 mm, the data variability is large
Sampson and Richards [18] Prospective cohort study Australia	47 (26/21) 8.91 (1.05) years 4 years	Leighton's method PM/M position *t* test, Pearson's correlation and multiple regression	G1	G2	G1	G2	G1	G2	Children with lower initial crowding (0.4 mm) may present a worsening of 0.6 mm ± 0.2 mm
			0.4 ± 0.4 mm	1 ± 0.45 mm	1 ± 0.45 mm	0.7 ± 0.35 mm	0.6 ± 0.2 mm	-0.3 ± 0.2 mm	
Hagberg [15] Prospective cohort study Sweden	24(14/10) 7 years (N.R) 6 years	Difference between incisor width and available space between deciduous canines Canine eruption Wilcoxon, Pearson and Spearman correlations	Male	Female	Male	Female	Male	Female	Mandibular incisor crowding tends to decrease in both sexes in the transition from mixed to permanent dentition after the eruption of permanent canines
			1.49 mm (N.R)	1.49 mm (N.R)	0.40 mm (N.R)	0.65 mm (N.R)	− 1.09 mm (N.R)	− 0.84 mm (N.R)	
Arslan et al. [17] Retrospective cohort study Turkey	65 (29/36) 9.54 (1.42) 5 years	Little index Intra/inter arch mx and md *t* test	1.31 (0.78)		1.11 (0.86)		− 0.19 (0.69)		Mandibular incisor crowding shows little self-correction in the dentition transition, related to leeway space and facial growth
Barros, et al. [16] Retrospective cohort study Brazil	42 (22/20) 8.66 (0.83) 4.58 (1.10) Years	Difference between incisor width and space between deciduous canines Cephalometric and intra-arch md measurements Wilcoxon, t test and multiple regression	G1 (≤ 2 mm) 0.73 (0.58)	G2 (≥ 2 mm) 3.96 (1.86)	G1 (≤ 2 mm) 0.61 (1.94)	G2 (≥ 2 mm) 1.46 (2.28)	G1 (≤ 2 mm) − 0.12 (1.89)	G2 (≥ 2 mm) − 2.50 (2.12)	The reduction in mandibular incisor crowding is related to the amount of initial crowding, leeway space, incisor protrusion, and increased maxillary arch width

*n* = sample, M = male, F = female, SD = standard deviation, G1 = group 1, G2 = group 2, mx = maxilla, md = mandible, PM = premolar, M = molar, N.R. = Not reported

### Assessment of risk of bias in individual studies

The risk of bias of the included studies was assessed with the Risk Of Bias In Non-randomized Studies of Exposure (ROBINS-E) [13]. Seven domains were considered including: (1) bias due to confounding; (2) bias in measurement of the exposure; (3) bias in participant selection; (4) bias in post-exposure intervention; (5) bias due to missing data; (6) bias in outcome measurement; (7) bias in the selection of reported results. The criteria adopted for the evaluation of each domain are described in Table 2. Again, risk of bias assessment was performed by both reviewers and disagreements were resolved by the third reviewer, if necessary.

**Table 2:** Criteria adopted for risk of bias assessment using Risk of Bias for in Non-randomized Studies of Exposures (ROBINS-E) tool

Domains	Criteria
Bias due to confounding	Studies are considered to be at **low risk** of bias if they consider the initial magnitude of mandibular incisor crowding, data on incisor protrusion, or information about the maxillary arch, including change in the width or amount of crowding. Studies are considered to be at **moderate risk** of bias if they consider the sex of individuals in the confounding factors. Studies are considered to be at **high risk** of bias if adjustment factors are not reported
Bias in measurement of the exposure	Studies are at **low risk** of bias if a valid way of measuring mandibular incisor crowding is reported. Studies are considered to be at **moderate risk** of bias if the measurement of incisor crowding is not performed on plaster or digital models. Studies are at **high risk** of bias if the way of measuring crowding was not reported
Bias in selection of participants	Studies are considered at **low risk** of bias if mandibular incisor crowding monitoring started concurrently with patient assessment, the exposed cohort has representativeness of the assessed exposure, or in the presence of a control group. Studies are considered at **moderate risk** of bias if the exposed cohort has some representation of the assessed exposure. Studies are considered at **high risk** of bias if there is no control group or if the exposed cohort does not faithfully represent the assessed exposure
Bias in post-exposure interventions	Studies are at **low risk** of bias if no intervention in the mandibular or maxillary arch was performed, while at **moderate risk** of bias if any intervention in the maxillary arch to attenuate crowding was performed. Studies are considered **high risk** if any intervention was performed on the mandibular arch, even without the objective of mitigating crowding
Bias due to missing data	Studies are considered at **low** risk of bias if less than 10% of participants were excluded to missing data, while at **moderate** risk of bias if less than 20%. Studies with higher proportion (≥ 20%) are considered at **high** risk of bias
Bias in outcome measurement	Studies are at **low risk** of bias if raters are not aware of the exposure level of mandibular incisor crowding or method error has been statistically evaluated. Studies are considered at **moderate risk** of bias if no reliability analysis has been performed in the intra-examiner assessment. Studies are at **high risk** of bias if the outcome assessment is based solely on self-report, without external validation
Bias in selection of reported results	Studies are at **low** risk of bias if all data planned by the authors in the entire sample are analyzed. Studies are considered at **moderate** risk of bias if they present outcome measures for only part of the sample population. Studies are at **high** risk of bias if all maxillary arch stability values ​​for all recommendations are not presented
Overall risk of bias	If at least one domain was found at **high** risk of bias, the overall risk was considered **high**. If at least one domain is at some concerns, but no domains are at high risk, the overall risk was considered **moderate**. If all domains were at **low** risk of bias, the overall risk was considered **low**

### Summary of measurements and synthesis of results

Initially, due to the continuous nature of the primary outcome (crowding changes in millimeters), the data were qualitatively selected in mean values and standard deviations. Based on these quantitative values, a meta-analysis was planned when the selected studies presented methodological, statistical, and clinical homogeneity of the data and the methods for obtaining it.

The included studies presented methodological differences in measuring crowding. Moreover, the studies presented some issues related to the risk of bias analysis. Overall, the increased between-study methodological heterogeneity would have generated unreliable information in the meta-analysis. Therefore, meta-analysis was not performed, and the results of the studies were qualitatively summarized and compared.

### Certainty of evidence

The certainty of evidence was assessed according to the Grading of Recommendations Assessment Development and Evaluations Pro software [14]. The narrative GRADE classified as not serious, serious, or very serious issues each one of the five domains evaluated: study design, risk of bias, inconsistency, indirectness, and imprecision of the articles. The final classification of the certainty of evidence was rated as high, moderate, low, or very low.

## Results

### Study selection

**Fig. 1:**
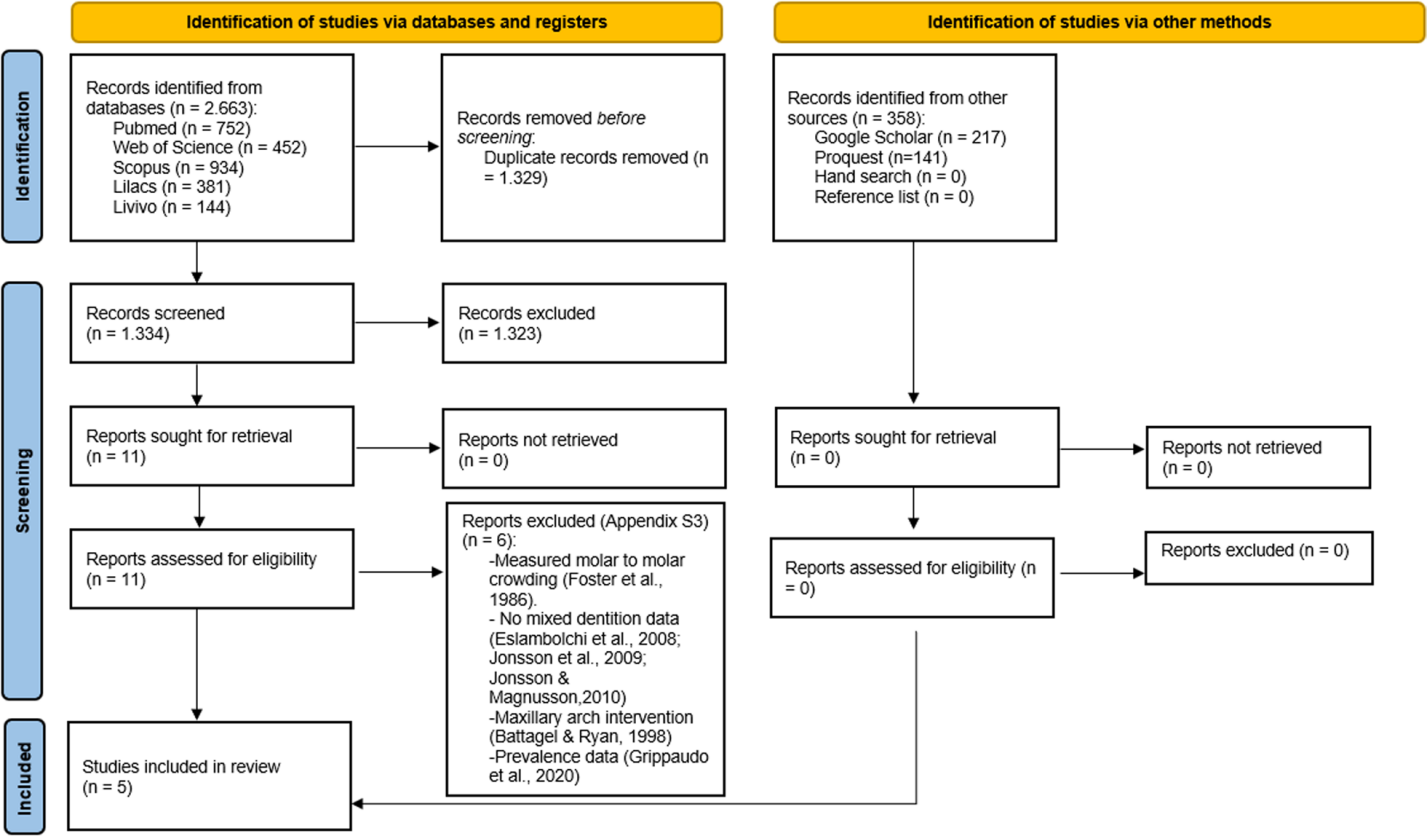
Flow diagram of study identification

The database searches identified 2.663 references: PubMed (*n* = 752), Web of Science (*n* = 452), Scopus (*n* = 934), LILACS (*n* = 381) and Livivo (*n* = 144). After duplicate removal, 1.334 studies remained. The gray literature search resulted in 358 references: Google scholar (*n* = 217) and ProQuest (*n* = 141). The hand search did not identify any potential study following the eligibility criteria. The reference lists of the included articles were evaluated in case of missing any relevant study. However no studies were identified. The titles and abstracts were screened, and 1.323 studies were discarded. Eleven studies were selected for full-text evaluation and applying the eligibility criteria. Of these, six were excluded with reasons. The references of excluded studies are reported in Additional file 3: Appendix S3, and the reasons for exclusion described in Fig. 1. Five studies were selected for qualitative synthesis [15–19]. The process of identification, screening, and exclusion of studies is described in the PRISMA flow diagram (Fig. 1).

### Study characteristics

The five studies were cohorts, two prospective [15, 18] and three retrospectives [16, 17, 19], including 243 patients: 124 males and 119 females. The mean initial age of patients ranged between 7 and 9 years [15, 19]. Three studies followed patients for an average of four years [16, 18, 19], one study followed for five years [17], and the longest follow-up was six years [15]. Three studies [16, 17, 19] evaluated the influence of dentofacial development on mandibular crowding in the mixed to permanent dentition. Another study evaluated the alignment of mandibular incisors before and after canine eruption [15]. Finally, a study evaluated possible predictors of mandibular incisor alignment in the mixed dentition [18].

Four studies evaluated dental models with dial caliper [15–17, 19]. One study performed measurements on photographed dental models [18]. To diagnose the outcome variable mandibular incisor crowding, two studies used the Little irregularity index [17, 19], two studies evaluated the difference between the width of the incisors and the space available between the deciduous canines [15, 16], and one study used the Leighton method [19]. Secondarily, intra- and inter-arch measurements were performed [16, 17, 19], cephalometric evaluations [16], association with the eruption of canines [15] and assessment of the position of unerupted posterior teeth [18]. The statistical analyses included *t* test [16–19], Pearson and Spearman correlations [15, 18, 19], Wilcoxon [15, 16] and multiple regression [16, 18]. The summary of data from the included studies is available in Table 1.

### Risk of bias in studies

**Fig. 2:**
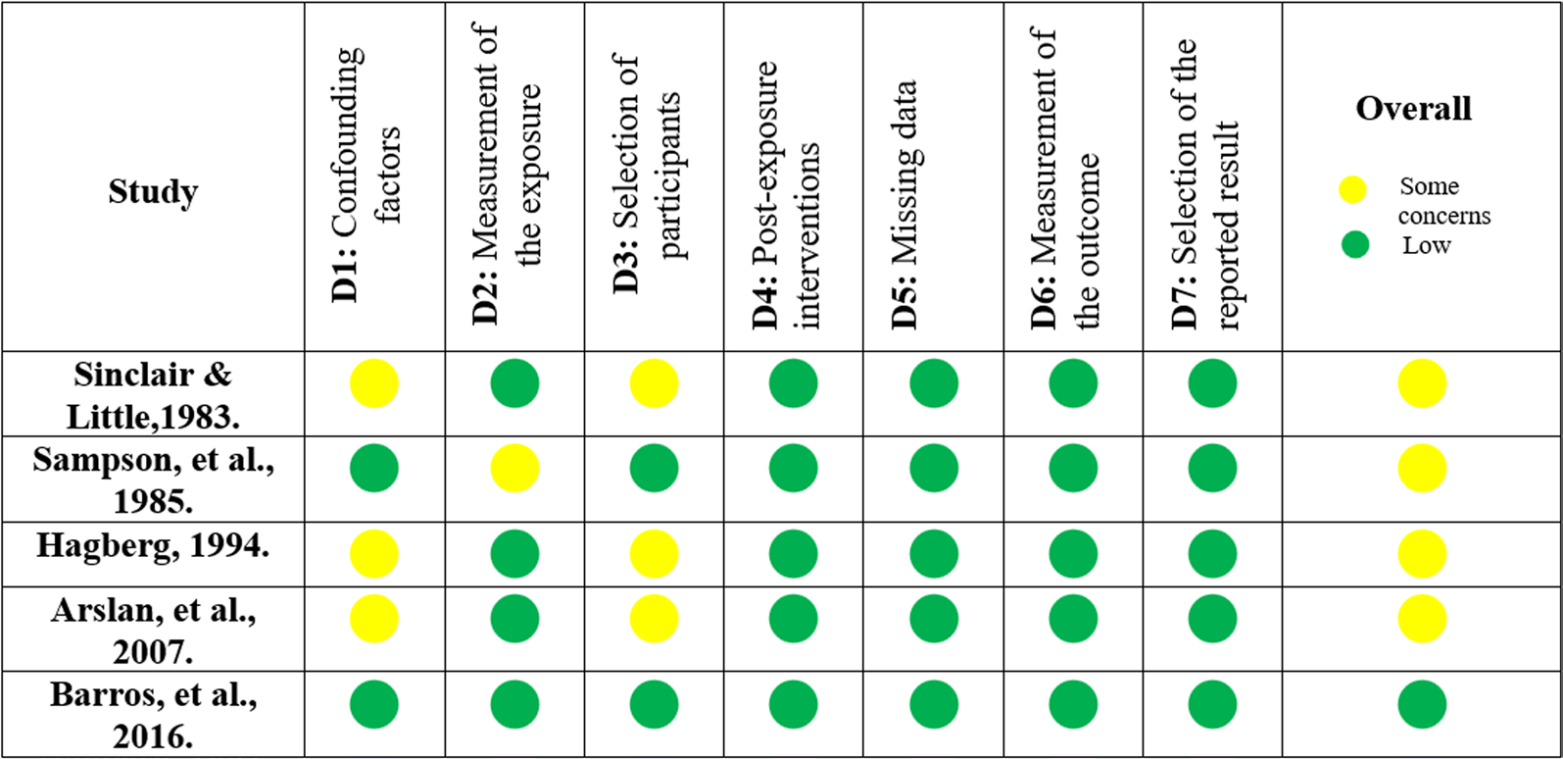
Risk of bias assessment using ROBINS-E tool

Among the five included studies, four presented moderate [15, 17–19] and one low risk of bias [16]. Some concerns related to the risk of bias were due to no control for confounding factors [15, 17–19] by studies that adjusted only the variable sex as a confounding factor in determining the results. Also, one study measured mandibular incisor crowding using photographed dental models, which introduces a risk of bias in the measurement of exposure [18]. Finally, three studies showed moderate risk of bias in the selection of participants, as they presented a partial representation of the evaluated exposure, considering the lower magnitude of crowding presented by the cohort at the beginning of the evaluation, less than 2 mm on average [15, 17–19]. The results of this risk of bias assessment are shown in Fig. 2.

### Results of individual studies

Retrospective studies with moderate risk of bias showed average crowding reductions of 0.17 (± 1.75) mm after 4-years follow-up using the Little irregularity index [19]. Differently, the prospective cohort with moderate risk of bias described crowding reductions from 0.3 to 0.6 (± 0.2) mm using the Leighton´s method [18]. The only study with a low risk of bias, which followed the sample for 4.58 ± 1.10 years, observed that children with an initial incisor crowding greater than 2 mm (3.96 ± 1.86 mm) had the greatest reductions in crowding until the permanent dentition (2.5 ± 2.12 mm). On the other hand, children with a mean mandibular incisor crowding of 0.73 ± 0.58 mm in the mixed dentition showed reductions of 0.12 ± 1.89 mm until the permanent dentition [16]. Likewise, the study that retrospectively evaluated children for five years with a mean value of mandibular incisor crowding in the mixed dentition of 1.31 ± 0.78 mm identified lower values of self-correction until the permanent dentition of 0.19 ± 0.69 mm. [17] Children prospectively followed in a study with moderate risk of bias for six years, who had a mean crowding of 1.49 mm in the mixed dentition, showed a mean self-correction of 0.96 mm in both sexes when evaluated in the permanent dentition [15].

### Certainty of evidence

The five included studies generated evidence with a moderate level of evidence regarding the reduction in mandibular incisor crowding in the transition from mixed to permanent dentition, even in children with an adequate occlusion. Considering the risk of bias domain was classified as serious due to the partial control of confounding factors by three studies [15, 17, 19]. Studies with a greater magnitude of mandibular incisor crowding in the mixed dentition [15, 16, 18] showed greater self-correction in the permanent dentition, indicating a dose–response gradient. The imprecision of the findings was what actually led to the classification of the certainty of the evidence as moderate. The evaluation of the evidence according to GRADE is described in Table 3.

**Table 3:** GRADE analysis

Certainty assessment							Impact	Certainty
No. of studies	Study design	Risk of bias	Inconsistency	Indirectness	Imprecision	Other considerations		
Reduction in mandibular incisor crowding from mixed to permanent dentition								
5	Observational studies	Serious	Not serious	Not serious	Serious	Dose–response effect detected	Studies reported a spontaneous mandibular incisor crowding reduction of 0.17 ± 1.75 to 2.50 ± 2.12 mm from the mixed to permanent dentition. The magnitude of crowding self-correction in the permanent dentition is directly related to the crowding severity in the mixed dentition. The greater the initial mandibular incisor crowding, the greater the spontaneous correction. Children with a crowding of 3.96 ± 1.86 mm in the mixed dentition had a reduction of 2.50 ± 2.12 in the permanent dentition. On the other hand, those that initially presented 0.7 ± 0.58 mm of mandibular incisor crowding in the mixed dentition presented a 0.12 ± 1.89 mm reduction in the permanent dentition	Moderate ⨁⨁⨁◯

## Discussion

Considering the five cohort studies included in this systematic review that followed children between 7 and 9 years of age for a period of 4 to 6 years without orthodontic interventions, moderate evidence was produced that mandibular incisor crowding tends to decrease in the transition from the mixed to the permanent dentition. That reduction is directly associated with the initial magnitude of incisor crowding. The greater the crowding in the mixed dentition, the greater its self-correction in the permanent dentition.

The only study that considered incisor crowding [16] as an eligibility criteria and examined children with initial average mandibular incisor crowding of 5.82 mm in the mixed dentition demonstrated that self-correction can reach up to 3.62 mm. It is speculated that greater crowding would stimulate a greater increase in intercanine distance [18, 20]. Even the other studies, which evaluated children with an adequate occlusion and little crowding [15, 17–19] or up to 1 mm of mandibular incisor crowding in the mixed dentition [18], presented a mean self-correction between 0.5 [18] and 1.92 mm [19] Thus, it is possible to identify a dose–response gradient, since the greater the crowding in the mixed dentition, the greater its ability to self-correct in the permanent dentition, even in children classified as having normal occlusion. The clinical recommendation to avoid orthodontic overtreatment in childhood and wait for the natural evolution of the occlusion seems opportune, unless crowding has a negative impact on child's quality of life and is greater than 4 mm in magnitude. It should be noted that due to the great variability of the data reported by the evaluated studies, a worsening of mandibular incisor crowding can also be expected [16]. Although children present approximately 2 mm of crowding in the mixed dentition, this does not exclusively mean that the prognosis is good [16]. Also, the initial amount of mandibular incisor crowding seems to justify, on average, 29–33% [18] of the self-correction. Therefore, the initial amount of crowding is not the only factor responsible for the self-correction, which shows the multifactorial etiology of this malocclusion.

Another important factor that should be considered in the spontaneous longitudinal changes of mandibular incisor crowding is the Nance´s leeway space, responsible for approximately 21% of the self-correction in the mandibular arch [16]. Contrary to these findings, a study with a moderate risk of bias reported that there is no correlation between leeway space and incisor or canine crowding, but the sample evaluated had an average crowding of 0.4–1 mm, therefore, a good occlusion [18]. The clinical implication of this small magnitude of crowding was considered in the use of the term good occlusion. Although some children may present little magnitude of crowding up to 1 mm, whose clinical implications may be questionable, they may have other characteristics of normal occlusion, such as positive horizontal and vertical overlap, Class I molar relationship, adequate inclination and angulation of dental crowns, among others. In statistical terms, stating that two variables are correlated does not mean that one can predict the other, as in logistic regression studies. Considering that the variability of data reported by all studies indicates that it is also possible to worsen crowding in the permanent dentition, the use of a lower lingual arch may be an alternative to be considered to avoid this worsening, especially if the child has more than 5 mm of crowding. A systematic review reports an improvement of up to 5 mm in mandibular incisor crowding with the use of the lower lingual arch, but the evidence generated has a very low level of certainty [21]. There is a need for randomized or controlled clinical trials to elucidate this issue.

Craniofacial growth also contributes to the self-correction of mandibular incisor crowding, as the mandibular anterior rotation that occurs in the mixed dentition leads to the protrusion of mandibular permanent incisors [22]. In addition, the greater proclination of the permanent incisors in relation to the deciduous predecessors also favors the self-correction of the mandibular incisor crowding [9]. In one of the studies evaluated [21], patients with a greater anterior mandibular rotation presented greater self-correction of crowding. Although the scope of this review does not include maxillary assessments, it is important to note that the increase in the width of the maxillary arch accounts for an average of 9% of the self-correction of lower incisor crowding [21]. The possible explanation is that width of the mandibular arch spontaneously accompanies the transverse increase in the arch. Maxillary [5], either physiological [21] or due to expansion [23].

Dentoalveolar factors participate expressively in the self-correction of mandibular incisor crowding in the transition from mixed to permanent dentition. After the eruption of permanent canines [5], the mandibular intercanine distance increases by an average of 3 mm [5]. Also, the eruption of the mandibular permanent incisors in line with the alveolar ridge works as a functional matrix stimulating the increase in dental arch width [9]. Thus, it does not seem adequate performing deciduous canine sttripping unless the incisors erupt outside the alveolar ridge line, usually lingual, characterizing persistent crowding. It might be an interesting therapy in economically disadvantaged regions, as it is cost effective. The literature indicates that the slice of deciduous canines is capable of eliminating up to 3 mm of crowding [24]. However, this systematic review demonstrates that this magnitude of crowding of up to 3 mm can be self-corrected in the transition of dentitions.

Regarding the sex characteristics of the children evaluated, this review does not indicate that there is a significant difference between men and women related to the amount of self-correction of mandibular incisor crowding in the permanent denture [15, 17]. This is evident even with a certain sexual dimorphism related to the size of the arches between men and women [25] which demonstrates smaller dimensions of the dental arches in women. One study [19] reported greater self-correction of mandibular incisor crowding in women, but when examining the result of a 0.2 mm improvement, it is clear that this magnitude is not clinically significant.

For the definition of clinical management in face of mandibular incisor crowding in the mixed dentition, it is important to consider the physiological mechanisms of occlusion compensation throughout the development of the mixed dentition [4, 5], as these may be sufficient to achieve self-correction. No therapeutic approach would do as much for the alignment of incisors without triggering side effects on the dental arch, as the mechanisms of compensation for the development of occlusion [9]. Thus, it seems reasonable that the clinician can consider the possibility of spontaneous alignment in magnitudes of approximately 4 mm of crowding. Although evidence was generated from observational studies, a dose–response gradient associated with the magnitude of crowding and its self-correction was detected. However, the imprecision detected between studies reduced the level of certainty of the evidence. This resulted in evidence with a moderate level of certainty and is extremely important for clinical practice. If there are any doubts on whether or not to intervene in crowding of mandibular incisors in the mixed dentition, it is interesting to consider the anatomical limitations of the mandibular arch related to molar distalization and arch expansion [26], impact on the child's quality of life, pain associated with orthodontic intervention, patient's socioeconomic status, and treatment time required to solve the incisor crowding.

### Limitations

The major question related to the limitations of this review refers to the sample selection of children with occlusion considered good even with a small initial magnitude of mandibular incisor crowding, by four [5, 22–24] of the five studies evaluated. However, even with this characteristic, a decrease in crowding was observed in all studies. Secondarily, the lack of primary studies and the lack of methodological rigor within some of these studies should be highlighted as another limitation of the evidence generated in this systematic review.

Several variables can explain the self-correction of crowding in the transition from mixed to permanent dentition. However, only two [16, 18] studies performed a logistic regression analysis to interpret the results looking for predictors of spontaneous changes in mandibular incisor crowding. The other studies [15, 17, 19] applied intra-group comparison or correlation tests. It is important to carry out studies that control for confounding factors, especially when trying to isolate the influence of growth factors from the alterations resulting from orthodontic interventions, such as the use of the lingual arch of Nance or the stripping of deciduous canines.

This review was performed from cohort studies. New randomized or controlled clinical studies may indicate changes resulting from orthodontic interventions to the detriment of those resulting from the child's craniofacial growth.

## Conclusions

- From five cohort studies, evidence was generated with a moderate level of certainty that mandibular incisor crowding reduces in the transition from mixed to permanent dentition by up to 4.62 mm, even in children with good occlusion. Also, the greater the mandibular incisor crowding in the mixed dentition, the greater the self-correction in the permanent dentition.
- In addition to the initial magnitude, other factors are associated with self-correction of childhood crowding, including leeway space, increased permanent incisor protrusion, and increased maxillary arch width.
- In orthodontic planning, the clinician can base their decision on the possibility of spontaneous alignment of mandibular incisor in childhood in children with crowding magnitudes up to 4 mm.
- It should be highlighted that the studies included in this review presented considerable variability. Thus, further prospective studies with greater methodological rigor may produce more accurate results and increase the level of certainty of the evidence.

## Supplementary information

**Additional file 1.** PRISMA checklist.

**Additional file 2.** Database search strategy.

**Additional file 3.** Reference list of excluded studies.

## Data Availability

The authors declare that all data generated or analyzed during this study are included in this published article and its supplementary information files.

## Declarations

### Protocol of the review

The review protocol was developed according to the updated Preferred Reporting Items for Systematic Reviews and Meta-Analyses (PRISMA 2020) statement.
