# Supplementary material for: Spontaneous changes in mandibular incisor crowding from mixed to permanent dentition: a systematic review
Source: Prog Orthod. 2023 May 8;24:15. doi: 10.1186/s40510-023-00466-3 (PMC10164666; doi:10.1186/s40510-023-00466-3)
Supplement: Supplementary file 2 — Additional file 2. Database search strategy. [file 40510_2023_466_MOESM2_ESM.docx]

**Appendix S2.** Database search strategy.

| **Database** | **Search strategy** | **Results** |
| --- | --- | --- |
| **PubMed** | ("dental change*"[Title/Abstract] OR "spontaneous change*"[Title/Abstract] OR "self correction"[Title/Abstract] OR "incidence"[Title/Abstract] OR "crowding reduction"[Title/Abstract] OR "permanent dentition"[Title/Abstract] OR "dentition, permanent"[MeSH Terms]) AND ("malocclusion"[MeSH Terms] OR "malocclusion*"[Title/Abstract] OR "crowding"[Title/Abstract] OR "crowding"[MeSH Terms] OR "incisor crowding"[Title/Abstract] OR "dental crowding"[Title/Abstract] OR "mandibular crowding"[Title/Abstract] OR "maxillary crowding"[Title/Abstract] OR "Little's irregularity index"[Title/Abstract]) AND ("children*"[Title/Abstract] OR "dentition, mixed"[MeSH Terms] OR "mixed dentition"[Title/Abstract] OR "transitional dentition"[Title/Abstract] OR "untreated"[Title/Abstract]) | **752** |
| **Web of Science** | **Topic (("dental change*" OR "spontaneous change*"OR "self correction" OR "incidence" OR "crowding reduction" OR "permanent dentition") AND TOPIC ("malocclusion*" OR "crowding" OR "incisor crowding" OR "dental crowding" OR "mandibular crowding" OR "maxillary crowding" OR "Little's irregularity index") AND TOPIC ("children*" OR "mixed dentition" OR "transitional dentition" OR "untreated")** | **452** |
| **Scopus** | ( TITLE-ABS-KEY ( ( "children*" OR "mixed dentition" OR "transitional dentition" OR "untreated" ) ) AND TITLE-ABS-KEY ( ( "malocclusion*" OR "crowding" OR "incisor crowding" OR "dental crowding" OR "mandibular crowding" OR "maxillary crowding" OR "Little's irregularity index" ) ) AND TITLE-ABS-KEY ( ( "dental change*" OR "spontaneous change*" OR "self correction" OR "incidence" OR "crowding reduction" OR "permanent dentition" ) ) ) | **934** |
| **Lilacs** | (( "children*" OR "mixed dentition" OR "transitional dentition" OR “dentición mixta” OR dentição mista OR dentadura mista” )) AND (("dental crowding" OR apinhamento OR “apiñamiento de dientes” )) AND ( ("self-correction" OR "spontaneous correction" OR “correção espontânea” OR “corrección espontânea” OR “crowding change” OR “dental change” OR “spontaneous change” OR “crowding reduction” OR “auto-correção”)) | **381** |
| **Livivo** | ("dental change*" OR "spontaneous change*"OR "self correction" OR "incidence" OR "crowding reduction" OR "permanent dentition") AND ("malocclusion*" OR "crowding" OR "incisor crowding" OR "dental crowding" OR "mandibular crowding" OR "maxillary crowding" OR "Little's irregularity index") AND ("children*" OR "mixed dentition" OR "transitional dentition" OR "untreated") | **144** |
| **Google Scholar** | "crowding" AND "mixed dentition" AND "spontaneous correction" " | **217** |
| **Proquest** | “Dental crowding” | **141** |
