# Supplementary material for: Spontaneous changes in mandibular incisor crowding from mixed to permanent dentition: a systematic review
Source: Prog Orthod. 2023 May 8;24:15. doi: 10.1186/s40510-023-00466-3 (PMC10164666; doi:10.1186/s40510-023-00466-3)
Supplement: Supplementary file 3 — Additional file 3. Reference list of excluded studies. [file 40510_2023_466_MOESM3_ESM.docx]

**Appendix S3.** Reference list of excluded studies.

1. Foster TD, Grundy MC. Occlusal changes from primary to permanent dentitions. Br J Orthod. 1986 Oct;13(4):187-93. doi: 10.1179/bjo.13.4.187. PMID: 3465367.
2. Grippaudo MM, Quinzi V, Manai A, Paolantonio EG, Valente F, La Torre G, Marzo G. Orthodontic treatment need and timing: Assessment of evolutive malocclusion conditions and associated risk factors. Eur J Paediatr Dent. 2020 Sep;21(3):203-208. doi: 10.23804/ejpd.2020.21.03.09. PMID: 32893653.
3. Battagel JM, Ryan A. Spontaneous lower arch changes with and without second molar extractions. Am J Orthod Dentofacial Orthop. 1998 Feb;113(2):133-43. doi: 10.1016/s0889-5406(98)70285-9. PMID: 9484204.
4. Eslambolchi S, Woodside DG, Rossouw PE. A descriptive study of mandibular incisor alignment in untreated subjects. Am J Orthod Dentofacial Orthop. 2008 Mar;133(3):343-53. doi: 10.1016/j.ajodo.2006.04.038. PMID: 18331931.
5. Jonsson T, Arnlaugsson S, Saemundsson SR, Magnusson TE. Development of occlusal traits and dental arch space from adolescence to adulthood: a 25-year follow-up study of 245 untreated subjects. Am J Orthod Dentofacial Orthop. 2009 Apr;135(4):456-62. doi: 10.1016/j.ajodo.2007.04.047. PMID: 19361731.
6. Jonsson T, Magnusson TE. Crowding and spacing in the dental arches: long-term development in treated and untreated subjects. Am J Orthod Dentofacial Orthop. 2010 Oct;138(4):384.e1-384.e7. doi: 10.1016/j.ajodo.2010.03.024. PMID: 20889033.
